# Rifampin Mono-Resistant Tuberculosis in New York City, 2010–2021: A Retrospective Case Series

**DOI:** 10.1093/ofid/ofad534

**Published:** 2023-11-21

**Authors:** Joseph A Lindsey, Alice V Easton, Herns Modestil, Felicia Dworkin, Joseph Burzynski, Diana Nilsen

**Affiliations:** Bureau of Tuberculosis Control, NewYork City Department of Health and Mental Hygiene, Long Island City, New York, USA; Bureau of Tuberculosis Control, NewYork City Department of Health and Mental Hygiene, Long Island City, New York, USA; Bureau of Tuberculosis Control, NewYork City Department of Health and Mental Hygiene, Long Island City, New York, USA; Bureau of Tuberculosis Control, NewYork City Department of Health and Mental Hygiene, Long Island City, New York, USA; Bureau of Tuberculosis Control, NewYork City Department of Health and Mental Hygiene, Long Island City, New York, USA; Bureau of Tuberculosis Control, NewYork City Department of Health and Mental Hygiene, Long Island City, New York, USA

**Keywords:** drug-resistant TB, rifampicin mono-resistance, rifampicin-resistant TB, tuberculosis

## Abstract

**Background:**

Although relatively rare, rifampin mono-resistant tuberculosis (RMR TB) poses important challenges to effective TB treatment and control. Information on the burden of RMR TB and treatment outcomes is needed to inform diagnosis and management.

**Methods:**

Standardized variables were collected from the New York City (NYC) tuberculosis surveillance system for patients treated for RMR TB in NYC during 2010–2021.

**Results:**

Of 7097 TB cases reported in 2010–2021, 31 (<1%) were treated clinically as RMR TB. Five (16%) of these patients had HIV. Seventeen patients (55%) had TB that was rifampin-resistant by both molecular and phenotypic drug susceptibility testing; 2 (6%) had rifampin resistance by phenotypic tests, and molecular tests were not done; and 12 (39%) were identified based only on molecular tests. Among these 12, 7 were rifampin-sensitive by phenotypic tests, and phenotypic testing could not be done for the other 5. Ten of the 31 (32%) were diagnosed in 2010–2015; the other 21 (including 10/12 diagnosed by molecular tests alone) were diagnosed in 2016–2021. Of the 31 patients, 21 (68%) completed treatment (median treatment duration of 18 months). Although the interval between tuberculosis treatment initiation and change to a non-rifamycin-containing regimen decreased significantly during the study period, the overall duration of treatment did not decrease significantly between 2010 and 2021.

**Conclusions:**

Molecular drug susceptibility tests identified cases of RMR TB that were not detected by phenotypic testing and helped enable timely adjustment of tuberculosis treatment regimens. Short-course regimens are needed to reduce duration of treatment for RMR TB.

Tuberculosis (TB) disease was the leading cause of death globally from a single infectious agent until the coronavirus pandemic in 2020 [1]. Rifampin (RIF) is the most effective first-line drug available for the treatment of TB, so resistance to RIF is of great concern [1]. RIF mono-resistant TB (RMR TB; resistance to RIF and isoniazid susceptibility) in the United States has been considered rare and associated with people with HIV (PWH) [2–6]. A study conducted between 1990 and 1997 in a public hospital in New York City (NYC) found that 81% of 21 patients who were treated for RMR TB were PWH [5], while another found that 79% of 96 NYC patients who were treated for RMR TB between 1993 and 1994 were PWH [6]. RMR TB may represent a growing and complicated threat for both PWH and others worldwide [2].

Although novel short-course treatment regimens for multidrug-resistant (MDR) TB have begun to improve outcomes and decrease duration of treatment [7–9], guidelines for the treatment of RMR TB have not been standardized [10]. Treatment regimens for patients with RMR TB are often lengthy [2, 11], and elevated rates of poor outcomes have been seen among patients with RMR TB [2, 11]. Regimens for RMR TB that are as short as possible, while still effective, could improve completion and cure rates for patients.

Testing for drug resistance has historically relied upon culture growth, sometimes resulting in delayed identification of resistance [12, 13]. The increase in use of molecular drug susceptibility testing (DST) has helped to hasten the discovery of RIF resistance in patients. One important molecular test is the Xpert MTB/RIF (Xpert) assay (Cepheid, Sunnyvale, CA, USA), approved by the US Food and Drug Administration (FDA) in 2013. This assay can identify *Mycobacterium tuberculosis* and mutations in the *rpoB* region of the *M. tuberculosis* genome (which can confer resistance to RIF) within hours of sputum collection [13–17].

When RMR TB is identified quickly, patients can be put on a new regimen promptly, avoiding prolonged treatment with an ineffective drug. A regimen targeted at RMR TB can help decrease symptoms more quickly, decrease the likelihood of TB transmission to others, and reduce the chance of TB relapse due to incomplete cure or the development of resistance to additional medications.

##  

### Aims

To understand the burden, scope, and trajectory of RMR TB in NYC, this study aimed to enumerate and describe patients with RMR TB between 2010 and 2021. To gauge progress in identifying and treating these patients, longitudinal trends in the time it took to identify and treat these patients were analyzed.

## METHODS

### Study Design and Data Collection

To quantify patients with RMR TB in NYC in 2010–2021, a retrospective case series was constructed. Patients were identified (and characterized in terms of demographic and clinical characteristics, diagnostic test results, and treatment regimens) using the NYC TB electronic disease surveillance and case management system (Maven 5.4., Conduent Inc., Florham Park, NJ, USA). Phenotypic DST methods included the Mycobacteria Growth Indicator Tube (MGIT; Becton Dickinson, Franklin Lakes, NJ, USA), solid agar proportion method, and minimum inhibitory concentration (MIC) testing. Molecular DST methods included the Xpert MTB/RIF (Xpert) assay (Cepheid, Sunnyvale, CA, USA), GenoType MDRTBplus (Hain LifeScience, Nehren, Germany), GenoType MDRTBsl (Hain LifeScience, Nehren, Germany), pyrosequencing [18], Sanger sequencing [19], and whole-genome sequencing.

### Definitions and Inclusion Criteria

RMR TB was defined as *M. tuberculosis* that is resistant to RIF but susceptible to isoniazid. In cases where there was inconclusive laboratory evidence, the classification as RMR TB relied on the judgment of the treating provider.

### Analysis

Linear regression was used to analyze trends in the incidence of RMR TB, the number of days between treatment start and the removal of RIF from the regimen, and the days between treatment start and treatment completion. The significance threshold used was .05. Analyses were done using R (version 3.5.2) and Microsoft Excel.

### Ethics Statement

The New York City Department of Health and Mental Hygiene (NYC DOHMH) Institutional Review Board determined that this project (Protocol #22-010) was exempt pursuant to 45 CFR §46.104(d)(4)(iii). This project involves only data routinely collected as part of normal program services.

### Patient Consent

Consent was not obtained for this secondary research analysis as the research activities only involved information that is regulated for public health purposes.

## RESULTS

### RMR TB in NYC, 2010–2021

**Figure 1.**
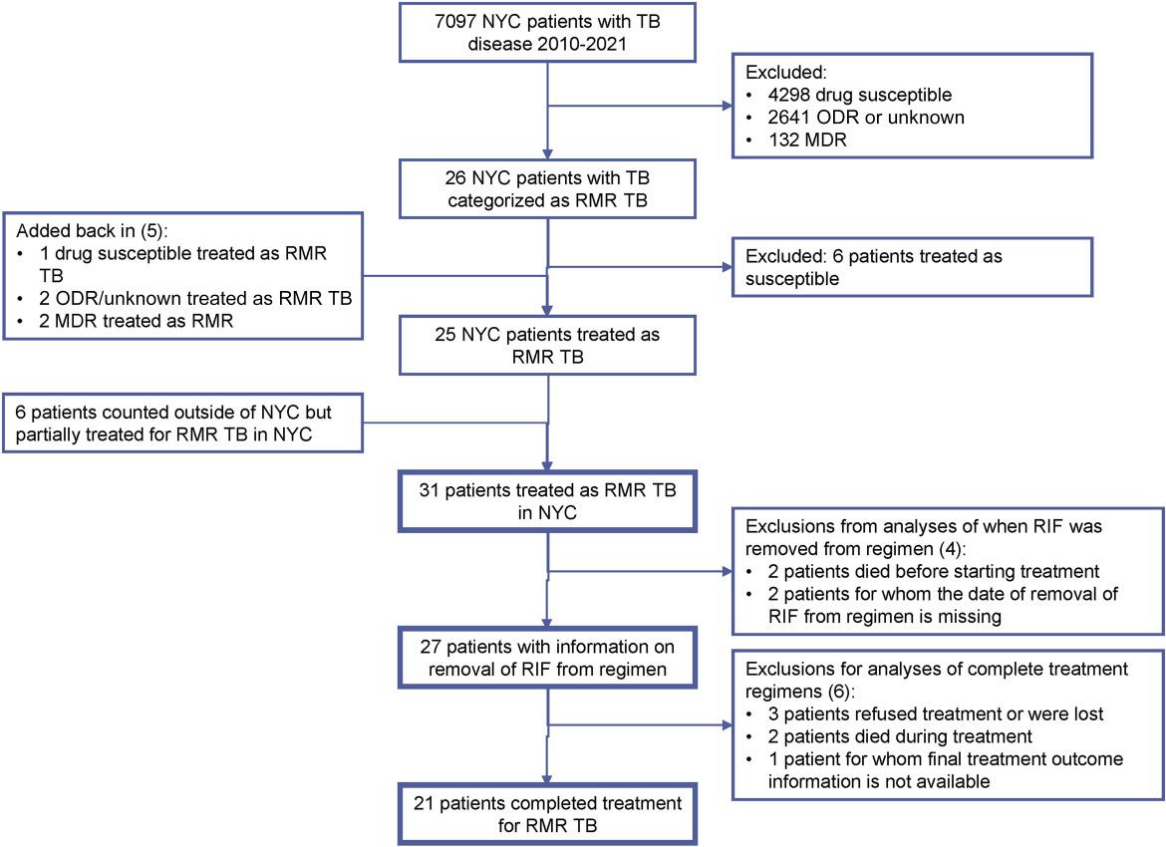
The set of 31 patients treated as having rifampin mono-resistant TB in NYC was defined based on sensitivity test results and clinical judgment. The 5 patients added and 6 patients excluded are described in the Supplementary Data. Some of these patients were excluded from analyses based on unavailability of relevant information. The key subsets analyzed in this study are the 31 total patients, 27 patients with information on when RIF was removed from their treatment regimen, and 21 patients who completed treatment for RMR TB. Abbreviations: MDR, multidrug resistance; NYC, New York City; ODR, other drug resistance; RIF, rifampin; RMR, rifampin mono-resistance; TB, tuberculosis.

Among 7097 patients diagnosed with TB in NYC from 2010 to 2021, 26 were categorized in the NYC TB electronic disease surveillance system as having RMR TB based on verifiable laboratory test results. Six of these patients were excluded from the study because they were treated for drug-susceptible TB based on clinical judgment (Supplementary Data). Five additional patients were included because they were treated by clinicians as having RMR TB, though they were categorized in the NYC TB electronic disease surveillance system as having drug-susceptible TB (n = 1), other drug resistance (n = 2), or MDR TB (n = 2). These patients are described in the Supplementary Data. An additional 6 patients were added to this case series because they were counted by the Centers for Disease Control and Prevention as part of the official case count of a jurisdiction other than NYC but were partially treated for RMR TB in NYC, for a total of 31 patients clinically determined to have RMR TB included in this study (Figure 1).

During the first 7 years (2010–2017) of the study period, RMR TB diagnoses accounted for <1% of all TB diagnoses in NYC (Supplementary Figure 1). Overall, between 2010 and 2021, there was a significant increase in the percentage of all TB cases counted in NYC that were categorized as RMR TB (*P* = .02) (Supplementary Figure 1). If the 12 cases that were only detected by molecular DST are removed, then there is no significant trend in the percentage of annual cases that were RMR TB (*P* = .17).

### Patient Characteristics

Among the 31 patients, 55% were men, the median age at diagnosis was 40 years, and 13% were born in the United States (Table 1). Most (22/31, 77%) had pulmonary TB, 7 (23%) had both pulmonary and extrapulmonary TB, and 2 (6%) had extrapulmonary TB alone (Table 1). Six (19%) had a history of TB. Twenty-nine (94%) had culture-positive TB. Five (16%) patients had HIV, and 5 (16%) patients had diabetes. Most patients (21, 68%) were treated at 1 of the 4 chest centers operated by the NYC DOHMH, and 24 (77%) participated in directly observed therapy during their treatment. One patient (patient 4 in Supplementary Table 1) had the same genotype as a patient diagnosed with TB in NYC in 2006, before the study period. Three patients (patients 15, 20, and 24) had the same genotype, though no definitive epidemiological links were found between these patients. One patient originally had pansensitive TB and acquired RIF resistance (patient 1), while another patient had RMR TB and later acquired isoniazid resistance (patient 15). Supplementary Table 1 shows demographic, clinical, and treatment information for each individual patient, listed chronologically by diagnosis date.

**Table 1.** Patients Clinically Determined to Have Rifampin Mono-Resistant TB in NYC, 2010–2021 (n = 31)

	No.	%
Gender		
Man	17	55
Woman	14	45
Age at diagnosis, median [IQR], y	40 [25–57]	…
Country of birth		
China	10	32
Ecuador	5	16
USA	4	13
Mexico	3	10
Other	9	29
Years in USA at time of TB diagnosis, median [IQR]^a^	6 [1–10]	…
History of TB	6	19
Site of disease		
Pulmonary	22	71
Extrapulmonary	2	6
Both	7	23
Initial chest x-ray^b^		
Normal	4	13
Abnormal noncavitary	20	64
Abnormal cavitary	7	23
Culture positive^c^	29	94
Diabetes	5	16
Living with HIV	5	16
Surgery for TB disease	1	3
Treatment outcome		
Treatment complete	21	68
Died	4	13
Moved	3	10
Refused	2	6
Lost	1	3
Ever on DOT	24	77
Treated in DOHMH clinic	21	68

Abbreviations: DOHMH, NYC Department of Health and Mental Hygiene; DOT, directly observed therapy; IQR, interquartile range; NYC, New York City; TB, tuberculosis.

^a^Based on 26 patients who were born outside the United States and had a record of the date they moved to the United States.

^b^This includes the 2 individuals with extrapulmonary TB only; 1 had a normal initial chest x-ray, and the other had an abnormal noncavitary chest x-ray (which was not consistent with TB).

^c^One person came to a chest center with a photo of Xpert results but no record of positive cultures from abroad (patient 16 in Supplementary Table 1). Another person (patient 28) had a lymph node pathology specimen that was sequenced by pyrosequencing and Sanger sequencing but never had a positive sputum culture.

### Diagnostic Methods

**Figure 2.**
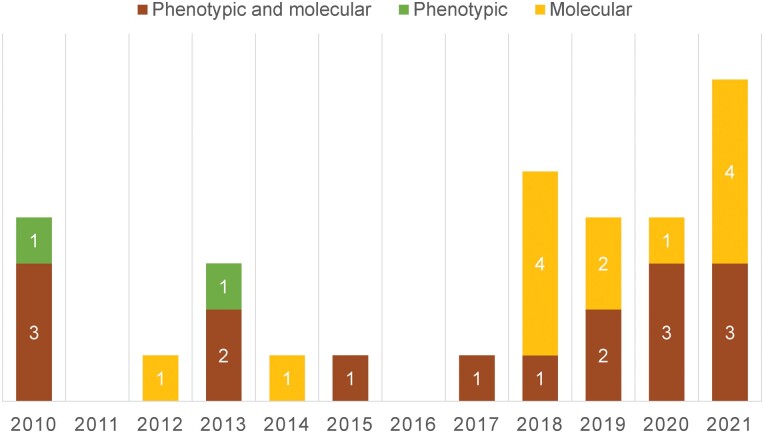
Method of diagnosis used to detect rifampin mono-resistant tuberculosis cases in New York City by year.

Over half of the patients (17, 55%) were diagnosed with RMR TB based on both phenotypic and molecular DST (Figure 2, Table 2). Twelve patients (39%) were diagnosed with RMR TB based on results from molecular DST alone, of whom 10 (83%) were diagnosed after 2015, when the Xpert test was widely adopted in New York State and New York City laboratories (Figure 2). In 7 of these 12 cases, phenotypic DST suggested that the isolate was susceptible to RIF, whereas in the remaining 5 cases phenotypic DST could not be done because a suitable specimen was not available (Table 2; Supplementary Data). Finally, 2 (6%) patients were diagnosed based on results from phenotypic DST alone, because molecular DST was not done (Figure 2, Table 2; Supplementary Data).

**Table 2.** Diagnosis of Patients With Rifampin Mono-Resistant TB (n = 31)

	Molecular DST: Resistant	Molecular DST: Not Done
Phenotypic DST: resistant	16 + 1^a^	2
Phenotypic DST: sensitive	7	0
Phenotypic DST: not done	5	0

Abbreviations: DST, drug susceptibility test; RMR, rifampin mono-resistant tuberculosis; TB, tuberculosis.

^a^Resistant to rifampin by minimum inhibitory concentration testing at 0.25 μg/mL, and whole-genome sequencing detected a low-confidence mutation. Patient technically categorized as pan-sensitive TB but treated as having RMR TB.

Twenty-six of the 31 patients had specific *rpoB* mutations identified (Table 3), 23 of whom had a mutation categorized by the World Health Organization (WHO) as being associated with RIF resistance [20, 21]. For the 5 people with the Leu452Pro mutation for whom phenotypic DST results were also available, phenotypic DST results suggested RIF sensitivity. Three mutations among the 31 patients are not on the WHO list (Table 3) [21].

**Table 3.** Mutations and Matched Phenotypic Drug Susceptibility Test Results for 31 Patients Clinically Determined to Have Rifampin Mono-Resistant TB

Mutation	WHO Resistance Group^a^	Patients in Study With Mutation	MGIT Drug Susceptibility Test Results	Conventional Drug Susceptibility Test Results
Arg528His	Not in catalogue	1	Not done	Not done
Glu266Ala	Not in catalogue	1	Sensitive	Sensitive
Gly534Glu	Not in catalogue	1	Resistant	Resistant
His526Cys	Associated with resistance	1	Not done	Not done
His526Leu	Associated with resistance	1	Resistant	Resistant
His526Tyr	Associated with resistance	5	5/5 resistant	5/5 resistant
Leu430Pro/Leu511Pro	Associated with resistance (previously borderline)	6	5/6 sensitive 1/6 not done	5/6 sensitive 1/6 not done
Leu452Pro/Leu533Pro	Associated with resistance	2	2/2 sensitive	1/2 resistant 1/2 not done
Pro520Leu	Associated with resistance—interim	1	Sensitive	Sensitive
Ser450Leu/Ser531Leu	Associated with resistance	7	6/7 resistant 1/7 not done	6/7 resistant 1/7 not done
Not available	Not applicable	5	3 resistant 2 not done	4 resistant^b^ 1 not done

Abbreviations: MGIT, Mycobacteria Growth Indicator Tube; TB, tuberculosis; WHO, World Health Organization.

^a^Official designations for each mutation according to the WHO's 2021 Catalogue of Mutations in *Mycobacterium tuberculosis* complex and their association with drug resistance [21].

^b^One phenotypic test result was of an unknown type; it may have been conventional, or it may have been another type of phenotypic test.

### Disease Outcome (n = 31)

Twenty-one patients (68%) completed treatment for RMR TB (Table 1). Of the 10 who did not complete treatment, 1 was lost to follow-up care, 2 refused treatment, 3 moved to other US jurisdictions outside of NYC soon after being clinically determined to have RMR TB, and 4 died (Supplementary Tables 1 and 2). Of the 4 patients who died, 2 were diagnosed with TB at the time of death, and 2 died during treatment. The 2 patients who died during treatment were on regimens that did not contain RIF when they died. One died less than a month after starting therapy (patient 10 in Supplementary Tables 1 and 2), and 1 died after ∼5 months of therapy (patient 23). All 4 had a cause of death that was related to TB and were over the age of 60 at the time of diagnosis. Among patients who started treatment, 72% (21/29) completed treatment.

### Trends in Time to Removal of RIF From Treatment Regimens (n = 27)

Information on when rifamycins were removed from the treatment regimens was available for 27 patients (Figure 1). There was a significant decrease in the time it took to remove rifamycins from the regimen between 2010 and 2021 (*P* = .01) (Supplementary Figure 2). The 5 patients who were never started on a rifamycin (removal at day 0) due to the rapid availability in later years of Xpert contributed to this trend. Two patients were on a rifamycin-containing regimen for >150 days, creating visible outliers in Supplementary Figure 2. One of these patients (patient 1 in Supplementary Table 1) was treated for pan-sensitive TB outside of NYC before RIF resistance was discovered, and the other (patient 27) was started on empiric treatment for TB and later produced a specimen that could be tested for resistance. Removing these 2 outliers did not alter the *P* value for these results.

### Trends in RMR TB Treatment Regimens Over Time

Analyses regarding drug regimens utilized for RMR TB, shown in Supplementary Table 2, were limited to patients who completed treatment (n = 21). Of these patients, 15 (71%) were started on isoniazid, RIF, pyrazinamide, and ethambutol (HRZE); 1 (5%) was started on HRZE, an injectable agent, and other second- and third-line medications; and the remaining 5 (24%) were never started on any form of HRZE combination therapy (Supplementary Table 2).

Fourteen of the 21 patients (67%) received an injectable agent during therapy. Ten of the 12 patients (83%) diagnosed in 2010–2019 received an injectable agent, all for >1 month (Supplementary Table 2). Only 4 of the 9 patients diagnosed after 2019 (44%) received an injectable agent, 3 for <1 month. Seventeen (81%) patients received a fluoroquinolone at some point during their treatment course, 16 of whom took a fluoroquinolone for >1 month.

Four patients completed therapy using the short-course all-oral 6- to 9-month regimen consisting of bedaquiline, pretomanid, and linezolid (BPaL) (Supplementary Table 2). In NYC, BPaL was first utilized by a patient who was treated for RMR TB beginning in 2018 using a combination of first- and second-line drugs, until the end of 2019, when additional specimens were collected that revealed the patient had acquired resistance to isoniazid, ethambutol, and pyrazinamide, classifying them as MDR (Supplementary Table 2). The patient was subsequently put on BPaL for an additional 6 months (Supplementary Table 2).

### Duration of Treatment (n = 21)

Among the 21 patients who completed treatment, the median treatment duration was 18 months (Supplementary Table 2). The median length of treatment was 20 months during 2010–2015 and 18 months during 2016–2021. Duration of treatment trended downward during the study period (Supplementary Figure 3). Although some patients completed treatment with BPaL, total treatment times were long among these patients due to the use of other anti-TB medications before the initiation of BPaL.

## DISCUSSION

The percentage of patients with TB in NYC who were clinically determined to have RMR increased between 2010 and 2021. Many of the recent diagnoses of RMR TB were made based on rapid molecular DST, which enabled prompt removal of rifamycins from treatment regimens. Despite these improvements, the duration of treatment for RMR TB remained long for most patients.

The increase in the proportion of patients with TB who were clinically determined to have RMR TB during the study period was likely due in part to an increase in the availability of molecular DST that can identify RMR TB, as shown by the high number of cases in later years that were identified by molecular DST alone. If the cases of RMR TB that were diagnosed based on molecular DST alone are removed, there was no significant increase in the percentage of yearly TB diagnoses that were RMR TB. A study in KwaZulu-Natal, South Africa, also found that the proportion of TB cases that were RMR increased from 15% to 21% between 2011 and 2014, a period during which Xpert became widely available for TB diagnosis there [22]. Though advancements in molecular diagnostic methods may have increased the ability to test for RMR, the number of patients who were treated for RMR TB (31) is still far fewer than the 96 patients who were treated for RMR TB in just 1993–1994 in NYC [6].

There are limited data concerning the prevalence of discordant molecular and phenotypic DST results and how best to manage patients with such results [23]. Several specific mutations are associated with discordant results (whereby it is not possible to detect resistance by conventional culture-based DST), but this is sometimes due to low-level resistance that can be seen by MIC testing and could influence patient outcomes [17, 24–27]. To discourage the use of RIF for patients whose mutations suggest that RIF may be ineffective for them, the WHO recommended that people whose TB infections had any of 7 *rpoB* mutations conferring “borderline resistance” be treated with a regimen for RIF-resistant TB, even if phenotypic DST suggests RIF sensitivity [28].

Early recognition of rifamycin resistance enabled several patients in this series to receive an initial treatment regimen that did not contain rifamycins. A major advantage of Xpert is that it can identify a mutation in the *rpoB* gene directly from a sputum sample, so rifamycin resistance can be recognized weeks before culture and subsequent phenotypic DST results are available [29–31]. Overall, there was a significant downward trend in the number of days between initiation of TB treatment and removal of rifamycins from treatment regimens during the study period. A large study of unique *M. tuberculosis* complex isolates at the New York State Wadsworth Laboratory found that molecular DST has resulted in resistance profiles being reported an average of 9 days earlier for first-line drugs (including RIF) when compared with conventional culture-based DST [13].

Patients with RMR TB often experience long treatment durations [2] and low treatment completion rates [2, 24, 32], and there is little guidance on treating RMR TB as separate from MDR TB [33]. In NYC, patients with RMR TB treated between 2010 and 2021 also experienced long treatment times, with a median length of treatment of 18 months. Similarly, a retrospective cohort study in South Korea found that patients with RMR TB treated between 1999 and 2013 had a median length of treatment of 15 months [32], and patients with RMR TB between 2000 and 2016 in Queensland, Australia, experienced a median length of treatment of 15 months [34].

Only 68% of patients in this study completed treatment. Similarly, 66% of patients with RMR TB treated in California between 1993 and 2008 completed treatment [3], and 51% of patients diagnosed in 2005–2010 in a retrospective cohort in France completed treatment [24]. In the study in South Korea, 77% of patients completed treatment [32]. Although similar to completion rates from these studies performed in different locations and time periods, the treatment completion rate among patients with RMR TB in our study was far below the 95.5% of all patients who completed treatment for TB in the United States in 2019 [35].

Four of the patients who completed treatment for RMR TB in NYC received the BPaL regimen, which received FDA approval for treatment of MDR and extensively drug-resistant TB in 2019 [36]. Since 2020, BPaL has been increasingly utilized as a first-choice regimen for patients with RMR TB in NYC. For most of 2010–2021, though less in recent years, injectable agents were a key part of regimens for RMR TB, and not just used during hospitalizations. Fluoroquinolones have also made up a key part of the regimens of nearly all patients in this cohort (Supplementary Table 2). Further utilization of BPaL as first-line treatment for RMR TB, and quicker procurement of the component medications, could lead to a decrease in treatment duration and an increase in treatment completion rates. One example of the potential for shorter regimens to lead to improved treatment success is that, among patients with RIF-resistant TB in a study in the Democratic Republic of the Congo, treatment success was associated with shorter regimens [31].

Historically, RMR TB has been associated with HIV coinfection [4, 5, 37]; 81% of 21 patients treated for RMR TB at a public hospital in NYC in 1990–1997 were PWH [5], and 79% of 96 NYC patients treated for RMR TB in 1993–1994 were PWH [6]. In contrast, only 5 (16%) patients diagnosed with RMR TB in NYC between 2010–2021 were PWH. Nonetheless, the proportion of patients who had HIV was higher in this case series than among all patients with TB in NYC during this period (8% in 2010 [38] and 5% in 2022 [39]). The linkage of RMR TB and HIV coinfection may have decreased over time in other parts of the United States, too; a retrospective cohort study in California found that the percentage of patients with RMR TB who had HIV coinfection decreased significantly from 1993 to 2008 [3]. A study of RMR TB in the United States (excluding California) from 1998 to 2014 also found that, while PWH were more likely to have RMR TB, the percentage of patients with RMR TB who had HIV declined over time [11]. These findings suggest that RMR TB is no longer limited to PWH in the United States and is instead increasingly being found among patients with TB regardless of whether they have HIV.

### Limitations

This study was based on programmatic data from a single geographical area. Each patient included in these analyses had a variety of factors that made their situation unique. Some information was missing for some patients, which necessitated the limitation of our data set for some analyses. Patients were included in the study sample if they were considered by the clinical team to have RMR TB, but some of these patients may not have truly had RMR TB. Patients with only Xpert results might have had undetected isoniazid resistance. In cases of discordance between tests, different clinicians may have made different treatment choices.

## CONCLUSIONS

Although RMR TB remains relatively rare, it has become more common in NYC since 2010 and is not limited to PWH. Molecular diagnostics have helped to identify an increased number of RMR TB cases in recent years. If molecular diagnostics had not been available, some of these patients would have been treated for rifamycin-susceptible TB and thus would likely have received a less effective regimen. Identifying RMR TB promptly enables initiation of appropriate treatment regimens, which should reduce the likelihood of onward TB transmission, speed symptom resolution, and reduce the risk of relapse and the development of resistance to additional medications. Xpert accelerated the discovery of RIF resistance in several patients in this case series. Despite these improvements, the duration of RMR treatment remained lengthy for patients in this study. This likely reflects the complexity of regimens that have been used in patients with RMR TB. As molecular DST becomes more advanced and widespread, the number of RMR TB cases that can be identified may continue to rise. The time necessary to complete effective treatment for RMR TB should decrease once there is more familiarity with short-course regimens and the procurement of the component medications is streamlined.

## Supplementary Material

ofad534_Supplementary_Data

## Supplementary Data

Supplementary materials are available at *Open Forum Infectious Diseases* online. Consisting of data provided by the authors to benefit the reader, the posted materials are not copyedited and are the sole responsibility of the authors, so questions or comments should be addressed to the corresponding author.
