# Supplementary material for: Rifampin Mono-Resistant Tuberculosis in New York City, 2010–2021: A Retrospective Case Series
Source: Open Forum Infect Dis. 2023 Nov 21;10(11):ofad534. doi: 10.1093/ofid/ofad534 (PMC10662657; doi:10.1093/ofid/ofad534)
Supplement: ofad534_Supplementary_Data [file ofad534_supplementary_data.pdf]

Rifampin mono-resistant tuberculosis in New York City, 2010-2021: a retrospective case series

Lindsey JA MPH BSPH[1]*, Easton AV PhD[1]*†, Modestil H BS[1], Dworkin F MD[1], Burzynski J MD MPH[1], Nilsen D MD[1]

Supplemental materials

Supplemental text

Supplemental text – Section 1

List of patients categorized as having rifampin (RIF) mono-resistant (RMR) tuberculosis (TB) but treated clinically as having drug susceptible TB (n=6). These patients are not among the 31 examined in this study.

1. Patient had an rpoB mutation according to a Xpert MTB/RIF (Xpert) assay (Cepheid, Sunnyvale, California) test, but all subsequent resistance tests suggested sensitivity and the patient was kept on a regimen containing RIF throughout their treatment.
2. Patient had an rpoB mutation according to a Xpert test, but all subsequent resistance tests suggested sensitivity and the patient was kept on a regimen containing RIF throughout their treatment (after a brief period where RIF was removed from the regimen).
3. Mutations were detected by multiple molecular methods, but the mutation was silent (Phe514Phe) and MGIT results categorized the isolate as sensitive.
4. Patient was resistant to RBT 0.5 based on conventional drug susceptibility testing (DST) in one lab, but sensitive based on results from another lab. The patient was sensitive to RIF based on all conventional DST results, no rpoB mutations were detected and RIF was not removed from the regimen.
5. Patient was resistant to RBT 0.5 based on conventional DST, but this was likely on a mixed culture. Conventional and MGIT DST found RIF-sensitivity and RIF was not removed from the regimen.
6. RIF resistant MGIT results were likely based on a mixed culture; subsequently, no rpoB mutation was found and conventional DST suggested pan-sensitivity.

[1]Bureau of Tuberculosis Control, New York City Department of Health and Mental Hygiene, 42-09 28th Street, Long Island City, NY 11101, USA

Supplemental text – Section 2

List of patients categorized as having other or unknown drug resistance patterns but treated clinically as having RMR TB (n=5):

1. ID 7: Patient was categorized as multi-drug resistant (MDR) based on a single result of isoniazid resistance on conventional DST. However, another conventional DST suggested isoniazid susceptibility, as did MGIT and pyrosequencing. The patient was kept on isoniazid throughout treatment.
2. ID 15: Patient was first categorized as having RMR TB and treated with a regimen containing isoniazid but not RIF. Isoniazid resistance was later acquired, and the patient was then treated on BPaL.
3. ID 16: No susceptibility testing was done in the US, and the patient was never culture-positive in the US. However, the patient came to a Chest Center with a photo of a lab report showing that an Xpert test identified an rpoB mutation.
4. ID 21: Patient was categorized as having pan-sensitive TB, because Xpert did not identify an rpoB mutation and MGIT and conventional DST found the samples to be RIF sensitive. However, whole genome sequencing detected an uncharacterized rpoB mutation (Glu266Ala) and a sample was resistant at 0.25 ug/ml by MIC DST.
5. ID 28: An rpoB mutation was detected, but the association of this mutation with RIF resistance was unknown. Sequencing probes for several other genes failed, so resistance to several other drugs was unknown. This person had extrapulmonary TB, and the sample was from a lymph node.

Supplemental text – Section 4

Why phenotypic DST wasn't done for 5:

1. ID 16: Never culture positive, as data not available. Presented to a Chest Center with a photo of Xpert results showing that an rpoB mutation was found.
2. ID 23: Mixed *Mycobacterium tuberculosis* and nontuberculous mycobacteria specimen. Whole genome sequencing was not possible, but Xpert and pyrosequencing were done.
3. ID 25: DST was only done by CDC's Division of Tuberculosis Elimination Laboratory Branch (Atlanta, Georgia) on a primary specimen.
4. ID 28: Never culture positive. Lymph node pathology specimen was TB positive by PCR. DST was done at the CDC's Division of Tuberculosis Elimination Laboratory Branch. Extrapulmonary TB only.
5. ID 13: Culture positive abroad, documented. Unknown phenotypic test used abroad.

Supplemental text – Section 5

Why molecular DST wasn't done for 2:

1. ID 8: Samples were taken in 2013 and molecular DST was unavailable.
2. ID 1: Samples were taken in 2009 and molecular DST was unavailable.

Supplemental Tables

Table S1: Case series of patients diagnosed with RMR TB in NYC, 2010-2021 (n=31)

ID	Age at Diagnosis	US-born?*	History of TB*	Initial Chest X-ray	Site of Disease	Culture	Diabetes	HIV status†	Treatment outcome	Ever on DOT?*
1	39	N	N	Normal	Pulmonary & Extrapulmonary	P	N	**P**	Treatment Complete	Y
2	20	N	N	Non-Cavitary	Pulmonary	P	N	N	Treatment Complete	Y
3	21	N	N	Non-Cavitary	Pulmonary	P	N	N	Treatment Complete	Y
4	25	N	N	Non-Cavitary	Pulmonary	P	N	N	Treatment Complete	Y
5	37	N	N	Normal	Pulmonary	P	N	**P**	Lost	Y
6	50	Unk.	N	Cavitary	Pulmonary	P	N	Unk.	Moved	Y
7	54	N	N	Non-Cavitary	Pulmonary & Extrapulmonary	P	N	**P**	Treatment Complete	**N**
8	24	N	N	Cavitary	Pulmonary	P	N	N	Treatment Complete	Y
9	21	N	N	Non-Cavitary	Pulmonary	P	N	N	Treatment Complete	Y
10	78	N	N	Non-Cavitary	Pulmonary	P	N	Unk.	Died	**N**
11	40	**Y**	**Y**	Normal	Pulmonary & Extrapulmonary	P	N	**P**	Treatment Complete	Y
12	22	N	N	Cavitary	Pulmonary	P	N	N	Refused	Y
13	23	N	N	Non-Cavitary	Pulmonary	P	N	N	Treatment Complete	Y
14	64	N	**Y**	Non-Cavitary	Pulmonary	P	N	N	Moved	Y
15	43	N	N	Non-Cavitary	Pulmonary	P	**Y**	Unk.	Treatment Complete	**N**
16	55	N	N	Non-Cavitary	Pulmonary	**N**	**Y**	N	Treatment Complete	Y
17	57	N	N	Cavitary	Pulmonary	P	**Y**	N	Treatment Complete	Y
18	35	N	N	Non-Cavitary	Extrapulmonary	P	N	N	Treatment Complete	Y
19	42	Unk.	N	Cavitary	Pulmonary	P	N	N	Moved	**N**
20	42	**Y**	N	Non-Cavitary	Pulmonary & Extrapulmonary	P	N	N	Treatment Complete	Y
21	65	N	N	Non-Cavitary	Pulmonary	P	N	**P**	Treatment Complete	Y
22	74	N	Unk.	Non-Cavitary	Pulmonary & Extrapulmonary	P	N	Unk.	Died	**N**
23	62	N	N	Non-Cavitary	Pulmonary	P	N	N	Died	**N**
24	32	**Y**	N	Non-Cavitary	Pulmonary	P	N	N	Treatment Complete	Y
25	76	N	**Y**	Cavitary	Pulmonary	P	**Y**	N	Died	**N**
26	52	N	**Y**	Non-Cavitary	Pulmonary	P	**Y**	N	Treatment Complete	Y
27	35	N	**Y**	Non-Cavitary	Pulmonary	P	N	Unk/Refused	Treatment Complete	Y
28	59	N	N	Normal	Extrapulmonary	P	N	N	Refused	Y
29	28	N	N	Cavitary	Pulmonary	P	N	N	Treatment Complete	Y
30	16	N	N	Non-Cavitary	Pulmonary	P	N	N	Treatment Complete	Y
31	35	N	**Y**	Non-Cavitary	Pulmonary & Extrapulmonary	P	N	N	Treatment Complete	Y

*Y – yes; N – no; Unk – unknown

†P – Positive; N – Negative; Unk. – Unknown

Table S2. Anti-TB medications used by patients who completed treatment (N=21)

ID	Length of Treatment (months)*	Initial Regimen†	Final Regimen†	First-Line Anti-TB Medications							Second-Line Anti-TB Medications						
				Rifampin	Isoniazid	Pyrazinamide	Ethambutol	Rifabutin	Injectables	Fluoroquinolones	Cycloserine	Ethionamide	Para-aminosalicylic acid	Clofazimine	Bedaquiline	Pretomanid	Linezolid
1	27	INH, RBT, PZA, EMB	EMB, ETH, LEV, CYC		<1 mo. ‡	<1 mo.	≥ 1 mo.	**<1 mo.**	≥ 1 mo.	≥ 1 mo.	<1 mo.	≥ 1 mo.					
2	18	HRZE	INH, EMB	≥ 1 mo.	≥ 1 mo.	≥ 1 mo.	≥ 1 mo.		≥ 1 mo.	≥ 1 mo.							
3	13	HRZE	INH, PZA, EMB, SMN	≥ 1 mo.	≥ 1 mo.	≥ 1 mo.	≥ 1 mo.		≥ 1 mo.								
4	10	HRZE	PZA, INH, EMB	≥ 1 mo.	≥ 1 mo.	≥ 1 mo.	≥ 1 mo.		≥ 1 mo.								
7	29	HRZE	INH, PZA, EMB, MOX	≥ 1 mo.	≥ 1 mo.	≥ 1 mo.	≥ 1 mo.		≥ 1 mo.	≥ 1 mo.							
8	20	HRZE	INH, PZA, EMB, LEV	**<1 mo.**	≥ 1 mo.	≥ 1 mo.	≥ 1 mo.		≥ 1 mo.	≥ 1 mo.							
9	20	HRZE	INH, PZA, EMB	≥ 1 mo.	≥ 1 mo.	≥ 1 mo.	≥ 1 mo.		≥ 1 mo.								
11	12	INH, RBT, PZA, EMB, MOX, LNZ, CYC, AMN, PAS	INH, PZA, EMB, LEV		≥ 1 mo.	≥ 1 mo.	≥ 1 mo.	**<1 mo.**	≥ 1 mo.	≥ 1 mo.	≥ 1 mo.		**<1 mo.**				≥ 1 mo.
13	18	AMN, PZA, LEV, RIF	PZA, INH, EMB	≥ 1 mo.	≥ 1 mo.	≥ 1 mo.	≥ 1 mo.			≥ 1 mo.		≥ 1 mo.		≥ 1 mo.			
15	21	INH, PZA, EMB, LEV	BPaL		≥ 1 mo.	≥ 1 mo.	≥ 1 mo.		≥ 1 mo.	≥ 1 mo.	<1 mo.	<1 mo.			≥ 1 mo.	≥ 1 mo.	≥ 1 mo.
16	11	INH, PZA, EMB, LEV	INH, PZA, EMB, LEV		≥ 1 mo.	≥ 1 mo.	≥ 1 mo.		≥ 1 mo.	≥ 1 mo.							
17	18	AMN, PZA, LNZ, EMB, LEV	INH, PZA, EMB, LEV		≥ 1 mo.	≥ 1 mo.	≥ 1 mo.		**< 1 mo.**	≥ 1 mo.	≥ 1 mo.				≥ 1 mo.		≥ 1 mo.
18	20	HRZE	INH, PZA, EMB, MOX	≥ 1 mo.	≥ 1 mo.	≥ 1 mo.	≥ 1 mo.			≥ 1 mo.							
20	13	HRZE	INH, PZA, EMB, LEV	≥ 1 mo.	≥ 1 mo.	≥ 1 mo.	≥ 1 mo.			≥ 1 mo.							
21	21	HRZE	INH, EMB, LEV	≥ 1 mo.	≥ 1 mo.	**<1 mo.**	≥ 1 mo.			≥ 1 mo.					≥ 1 mo.		≥ 1 mo.
24	17	HRZE	INH, PZA, CYC, MOX	**<1 mo.**	≥ 1 mo.	≥ 1 mo.	≥ 1 mo.		≥ 1 mo.	≥ 1 mo.	≥ 1 mo.						
26	17	RIF, PZA, EMB, LEV	BPaL	≥ 1 mo.		≥ 1 mo.	≥ 1 mo.		**<1 mo.**	**<1 mo.**					≥ 1 mo.	≥ 1 mo.	≥ 1 mo.
27	18	HRZE	INH, PZA, EMB, MOX	**<1 mo.**	≥ 1 mo.	≥ 1 mo.	≥ 1 mo.			≥ 1 mo.							
29	9	HRZE	BPaL	≥ 1 mo.	≥ 1 mo.	≥ 1 mo.	≥ 1 mo.								≥ 1 mo.	≥ 1 mo.	≥ 1 mo.
30	19	HRZE	EMB, LEV, LNZ	≥ 1 mo.	≥ 1 mo.	≥ 1 mo.	≥ 1 mo.			≥ 1 mo.							≥ 1 mo.
31	14	HRZE	BPaL	≥ 1 mo.	≥ 1 mo.	≥ 1 mo.	≥ 1 mo.		**<1 mo.**	≥ 1 mo.					≥ 1 mo.	≥ 1 mo.	≥ 1 mo.

*Total treatment time for all patients, including those who completed short-course regimens, includes any time spent on a holding regimen, a regimen for pansensitive TB prior to the discovery of drug resistance, or while treatment was being held or a patient was lost after having started treatment, prior to the successful completion of treatment.
†RIF – rifampin; INH – isoniazid; PZA – pyrazinamide; EMB – ethambutol; RBT – rifabutin; LEV – levofloxacin; MOX – moxifloxacin; CYC – cycloserine; AMN – amikacin; LNZ – linezolid; PAS – para-aminosalicylic acid; HRZE – isoniazid, rifampin, pyrazinamide, ethambutol; BPaL – bedaquiline, pretomanid, linezolid
‡Total months on specified drug

Supplemental Figures

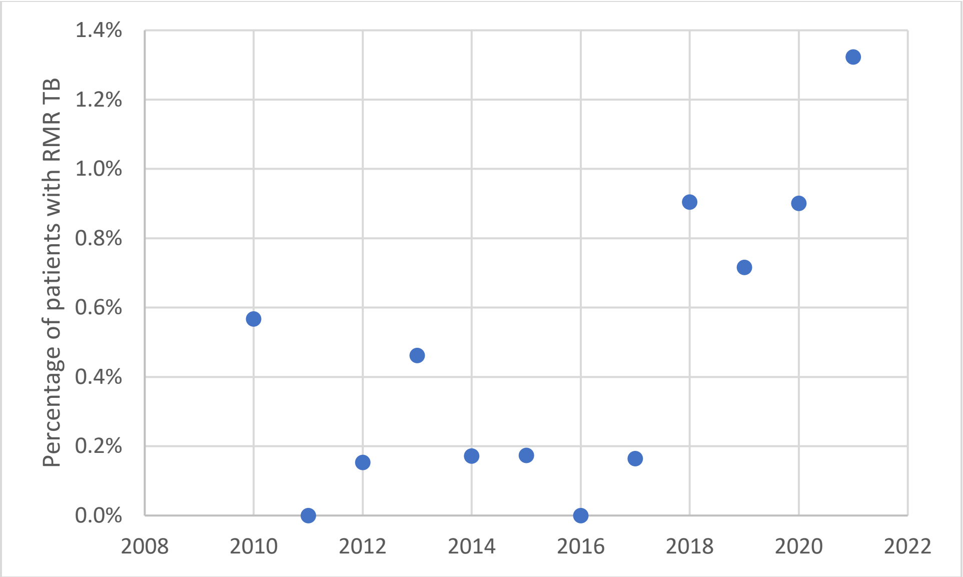

Figure S1. The percentage of total patients diagnosed with TB in NYC, by year, who had rifampin mono-resistant TB. There was a significant increase during this period (based on 31 patients, p=0.02).

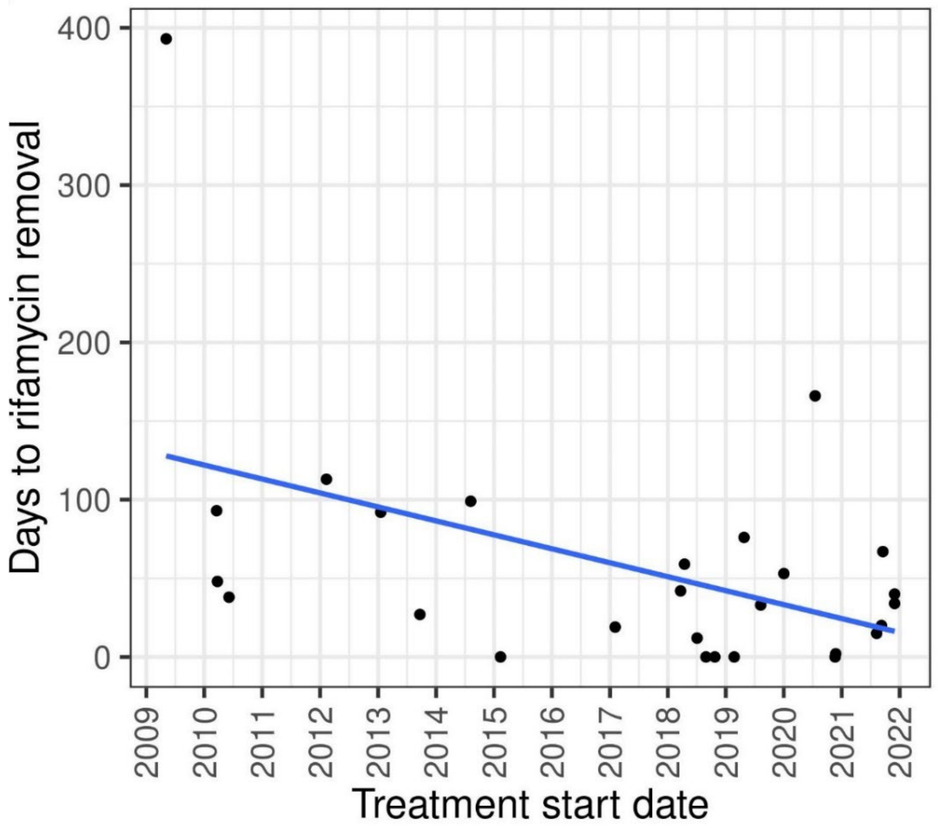

Figure S2. For patients with sufficient information about the removal of rifampin from the regimen (n=27) the number of days between treatment start and the removal of rifampin from the regimen is plotted against the treatment start date.

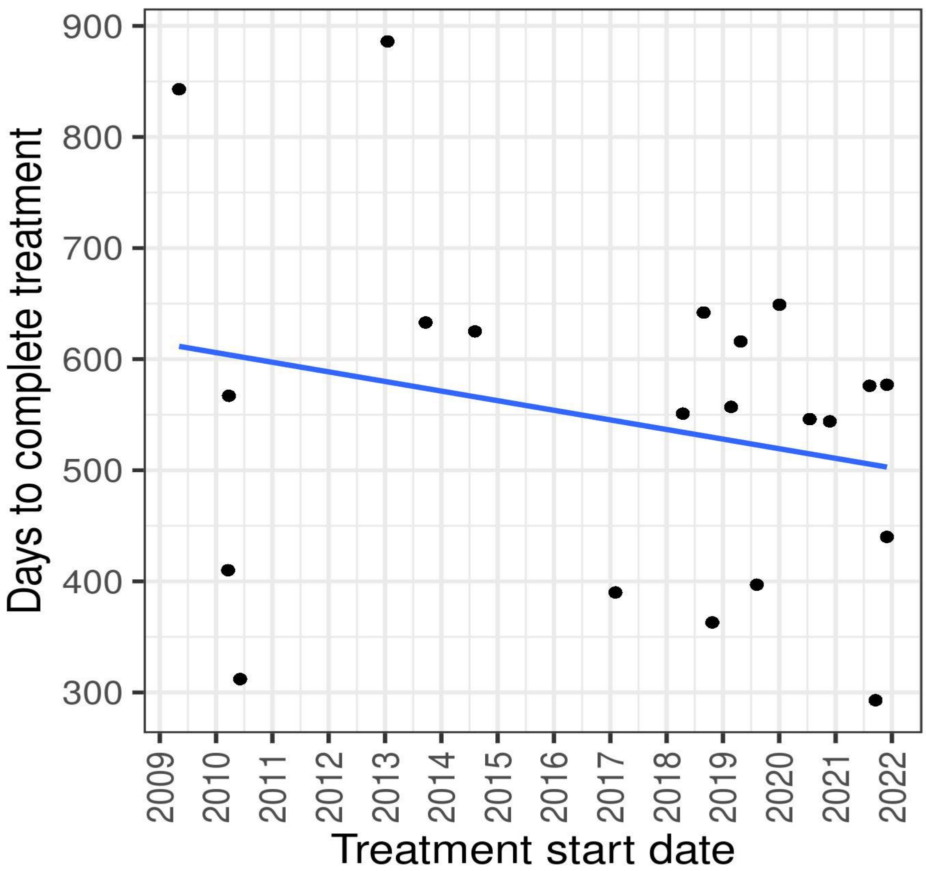

Figure S3. For patients who completed treatment for rifampin mono-resistant TB (n=21) the number of days between treatment start and completion is plotted against the treatment start date.
